# Metabolic and Reproductive Responses to Peripartum Feed Supplementation in Hyperprolific Gilts

**DOI:** 10.3390/life16030416

**Published:** 2026-03-04

**Authors:** Julia Cantin, Carlos Cantin, Olga Mitjana, Maria Teresa Tejedor, Carlos Gil-Rubio, Ana Maria Garrido, Maria Victoria Falceto

**Affiliations:** 1 Department of Animal Pathology, University of Zaragoza, 50013 Zaragoza, Spain; jcantin@unizar.es (J.C.); omitjana@unizar.es (O.M.); amgarrido@unizar.es (A.M.G.); vfalceto@unizar.es (M.V.F.); 2 Independent Researcher, 50009 Zaragoza, Spain; carlos@agnporcino.com; 3 Agroalimentary Institute of Aragon-IA2, Department of Animal Pathology, Universidad de Zaragoza-CITA, 50013 Zaragoza, Spain; 4 Department of Anatomy, Embryology and Animal Genetics, CIBERV, University of Zaragoza, 50013 Zaragoza, Spain; 5 Veterinary Technical Service Nutega, CCPA Group, 28823 Coslada, Spain; cgil@nutega.com

**Keywords:** feed supplementation, gilts, peripartum, reproduction, metabolism

## Abstract

Gilts have a lower capacity for voluntary feed intake and body reserves than multiparous sows, which limits their ability to cope with the needs of gestation and lactation. In this study, a nutritional supplement was formulated to support gilts during the peripartum period. Both control (C, n = 64) and treatment (T, n = 63) groups received standard commercial diets. Group T received 300gr of supplement per gilt and day for the last 35 days of gestation until the fifth day of lactation. This supplement contained calcium (Ca; 4.1%), sodium (Na; 4.0%), lysine (Lys; 1.96%), methionine (Met; 1.32%), vitamin B_12_ (0.3 mg/kg), choline chloride (600 mg/kg), betaine (475 mg/kg), and L-carnitine (500 mg/kg). Supplementation significantly reduced (*p* < 0.050) stillbirth rate, neonatal diarrhea, postpartum hypophagia, and both β-hydroxybutyrate (BHBA) and creatinine (CREA) concentrations (effect sizes: 0.240–0.993). Also, supplementation significantly increased (*p* < 0.050) piglet weight at birth and at 15 days of lactation and maternal backfat thickness at 26 days of lactation (effect sizes: 0.491–0.719). The concentrations of BHBA and CREA showed significant and negative associations with several productive parameters (*p* < 0.05); the strength of the associations was low–medium. Targeted peripartum supplementation represents a feasible nutritional strategy for commercial herds characterized by large litter sizes and limited voluntary feed intake capacity.

## 1. Introduction

Genetic selection for hyperprolific sows has substantially increased litter size over recent decades, representing a major achievement in modern pig production [1,2,3]. However, this progress has been accompanied by important biological and management challenges, particularly in nulliparous sows. Large litters are frequently associated with reduced piglet birth weight, increased within-litter variability, compromised neonatal vitality, and lower growth rates during lactation, ultimately resulting in reduced weaning weights and higher pre-weaning losses [4,5,6]. Compared with multiparous sows, gilts typically exhibit lower voluntary feed intake capacities, reduced body reserves, and less developed mammary tissue, which further limits their ability to meet the nutritional demands of pregnancy and early lactation [7,8,9].

In modern sow populations, genetic improvement has primarily targeted lean tissue accretion and feed efficiency, traits closely linked to reproductive physiology and metabolic regulation [10]. In nulliparous sows, adequate nutrition must simultaneously support ongoing somatic growth, fetal and placental development, and preparation for lactation. Insufficient nutrient supply during this period can impair colostrum synthesis, prolong farrowing, increase stillbirth rates, and compromise early lactational performance [11,12,13]. Consequently, nutritional management during late gestation and early lactation represents a critical determinant of both short- and long-term sow productivity.

The peripartum or transition period—encompassing the final days of gestation and the first days of lactation—is characterized by profound physiological, endocrine, and behavioral changes [14,15,16]. During this stage, sows experience abrupt shifts in feed intake, housing, and endocrine regulation, while fetal growth accelerates and colostrum and milk synthesis commence. In hyperprolific gilts, these demands frequently exceed voluntary feed intake capacity, increasing the risk of negative energy balance and metabolic stress [9,17,18].

Extensive mobilization of body reserves is commonly observed during late gestation and early lactation in hyperprolific gilts. Protein catabolism from skeletal muscle and visceral tissues may impair farrowing performance, maternal recovery, and subsequent reproductive cycles [7,19]. In parallel, insufficient energy intake promotes lipid mobilization and ketone body production, reflecting impaired metabolic adaptation to the peripartum period [20,21,22,23]. These metabolic disturbances may also compromise immune function and colostrum quality, thereby increasing the susceptibility to neonatal disorders and early piglet mortality [24,25,26,27].

Beyond macronutrient supply, the peripartum period is associated with increased oxidative stress and altered mineral homeostasis. Replacement gilts often exhibit higher oxidative stress markers than multiparous sows, likely reflecting lower metabolic resilience and antioxidant capacity [28,29,30]. Disturbances in calcium homeostasis around parturition may impair uterine contractility, prolong farrowing, and increase the risk of stillbirths [31,32,33,34]. Collectively, these challenges highlight the need for targeted nutritional strategies that support overall metabolic stability during the transition period.

Traditional nutritional interventions during late gestation have focused on increasing feed allowance (“bump feeding”) to improve fetal growth and prepare sows for lactation [13,18]. However, the effectiveness of such strategies may be limited in hyperprolific gilts due to reduced feed intake capacity and the complexity of nutrient demands during the peripartum period. Consequently, targeted multi-nutrient supplementation strategies that enhance overall nutrient supply without substantially increasing feed bulk have gained interest under commercial conditions [9,17,35].

Therefore, the objective of the present study was to evaluate the effects of a targeted peripartum multi-nutrient supplement administered during late gestation and early lactation on metabolic status, reproductive performance, and early litter growth in hyperprolific gilts. We hypothesized that improving overall nutrient availability during the peripartum period would enhance metabolic adaptation, reduce catabolic stress, improve farrowing outcomes, and support early piglet performance.

## 2. Materials and Methods

The authors declare that they have not used any type of GenAI during any part of this work (the generation of text, data, or graphics, or assistance in study design, data collection, analysis, or interpretation).

All protocols used in this study were approved by the Animal Ethics Committee of the University of Zaragoza (reference number: PI37/23; date: 29 July 2024). This study complied with ARRIVE guidelines [36]. The procedure was carried out on a commercial farm in north-eastern Spain. Expert veterinarians supervised the care and handling of the animals, ensuring their welfare throughout the experiment.

### 2.1. Experimental Design, Housing, and Management

This study included 127 nulliparous sows (DanBred Landrace × DanBred Large White) divided into control (C, 64 sows) and treatment (T, 63 sows) groups. Only nulliparous sows were included in the study and housed separately from multiparous sows throughout gestation and lactation. Gilts were randomly assigned to groups and housed in pens during gestation, transitioning to individual farrowing crates from day 110 of gestation to 28 days postpartum. This study was conducted over seven months (April–October 2025) on a farm with 620 reproductive sows and ensured that all gilts belonged to the same contemporary group.

Estrus was monitored daily, and gilts were inseminated with a commercial semen dose (2 × 10^9^ sperm cells); a second insemination was performed after 24 h if estrus persisted. Group T received a nutritional additive supplement starting on day 80 of gestation until five days postpartum, in addition to the basal diet. Control gilts were fed only the basal diet, which was formulated according to the National Research Council recommendations [37].

Gilts entered the farm at five months of age (approximately 100 kg body weight) and were acclimated in pens of 25 gilts for 8 weeks with ad libitum feeding. Subsequently, the largest gilts were allocated to weekly batches of six animals and fed a commercial lactation diet with flushing and altrenogest treatment for estrus synchronization. Pregnancy was confirmed by ultrasonography 24 days after insemination.

Gestating gilts were housed in pens of 55 animals with partially slatted floors, providing 3 m^2^ per gilt, and were fed using semi-stall-feeding systems. Feeding commenced at 2.5 kg/day of the basal diet and continued until farrowing, with individual confinement for 30 min daily from day 80 of gestation to allow for controlled feeding. Farrowing crates (1.80 × 2.65 m) were equipped with individual drinkers (water flow rate of 3 L/min), creep areas with heated floors, and infrared lamps for piglets. Farrowing occurred naturally, with obstetrical assistance provided when birth intervals exceeded 20 min. Litters were standardized within 24 h postpartum to 13–14 piglets per sow, matched for piglet size and litter number across experimental groups.

### 2.2. Dietary Treatments, Feeding, and Feeding Systems

Two feeding diets were designed and randomly assigned to the control group (C) and the treatment group (T) in this experiment. Blinding was not feasible under commercial farm conditions, as farm staff were aware of group allocation due to feed management logistics.

#### 2.2.1. Control Group (C)

The control group was fed a commercial diet formulated according to recommendations from the Spanish Foundation for the Development of Animal Nutrition (FEDNA) for nutritional tables for production sows [38], which are based on established nutrient requirement guidelines for swine [37]. Table 1 illustrates that the feeding program consisted of a gestation diet administered from weaning until day 110 of gestation and a lactation diet provided from day 110 of gestation until 28 days postpartum. Feeding followed a “flat” curve of 2.5 kg/day from mating until day 110 of gestation, after which the feeding strategy was adjusted to match that of multiparous sows, as described by Cantin et al. [7].

#### 2.2.2. Treatment Group (T)

Group T was fed the same commercial diet as group C, formulated according to the FEDNA recommendations for swine nutrition [38]. Table 1 illustrates that the feeding program consisted of a gestation diet administered from weaning to day 110 of gestation and a lactation diet provided from day 110 of gestation to 28 days postpartum. The supplement was administered at 300 g per sow per day during the final 35 days of gestation, as part of a single daily feeding regimen, and during the first 5 days of lactation during morning feeding, following the protocol described by Cantin et al. [7]. The supplement was provided individually during controlled feeding periods, ensuring that no feed-stealing occurred between sows.

The supplement composition was carefully designed to optimize maternal and fetal health:

- Electrolyte Balance: The electrolyte balance was adjusted to approximately 200 mEq/kg using Mogin’s formula [39]:

Balance = mEq/kg Na + mEq/kg K − mEq/kg Cl = Na × 434.97 + K × 255.74 − Cl × 282.06.

Sodium was added to achieve the desired balance. This ensured the partial balance of cations and anions in the diet, which is of great importance in metabolism due to its participation in osmotic balance, acid–base balance, and the integrity of the mechanisms that regulate transport across cell membranes.

- Calcium: Calcium was included in sufficient amounts to mitigate hypocalcemia during the peripartum period, which is caused by demineralization during late gestation. Phosphorus levels were also balanced.
- Lysine and Methionine: These amino acids were included to meet muscle growth needs in sows and the development of fetal and placental tissues, thereby helping to prevent muscle loss and ketosis.
- Vitamins and Provitamins: Vitamin B_12_, choline chloride, betaine, and L-carnitine were incorporated for their hepatoprotective effects.
- Ingredients: The supplement contained extruded wheat, dehulled soybean meal (genetically modified organism), calcium carbonate, and powdered whey.
- Analytical Components: Calcium, 4.1%; sodium, 4%; phosphorus, 0.3%; lysine, 1.96%; and methionine, 1.32%.
- Additives per Kilogram of Supplement:
  ◦ Vitamins: Vitamin B_12_ (0.3 mg), choline chloride (600 mg), betaine anhydrous (475 mg), and L-carnitine (500 mg).
  ◦ Trace Elements: Selenomethionine (from Saccharomyces cerevisiae) (1.1 mg).
  ◦ Preservatives: Citric acid (E330) (209.5 mg) and sodium propionate (E282) (150,000 mg).
  ◦ Antioxidants: Butylated hydroxytoluene (BHT, E321) (166.5 mg) and propyl gallate (E310) (166.5 mg).
  ◦ Anti-Caking Agents: Sepiolite (E562) (100 g) and precipitated and dried silica (E551a) (7.8 g).
  ◦ Flavoring Agents: Flavoring mix (1.053 mg).

The supplement was produced as a dry meal-type additive with a particle size typical of commercial feed additives (generally <1 mm), ensuring adequate flowability and minimizing segregation. The supplement was manually distributed by trained farm staff during controlled individual feeding periods on top of the daily ration, ensuring accurate individual intake and preventing feed-stealing. Homogeneity was ensured through standardized mixing procedures during manufacturing, and given the on-top administration and individual feeding management, within-batch homogeneity was considered adequate for the objectives of this study.

For each batch of gestation and lactation feed delivered to the farm, a representative 1 kg sample was collected on arrival, placed in a sealed plastic bag, and submitted for analysis. Feed samples were analyzed using near-infrared reflectance spectroscopy (NIRS) in a commercial laboratory routinely used for feed evaluation. This approach allowed us to verify that the nutrient levels of the delivered feeds were consistent with formulation specifications throughout the experimental period.

### 2.3. Data Collection and Chemical Analysis

#### 2.3.1. Body Condition

Body condition was assessed by measuring backfat thickness (BFT) and longissimus muscle depth (LMD) at the P2 reference point, 6 cm from the midline and behind the last rib, using a wireless ultrasound scanner (Backfat & Loin Depth Scanner SF-1, SonicVet, Beijing, China) with a 5 MHz linear digital probe connected to an iPad via Wi-Fi. These measurements were collected for all animals in both groups on days 80 and 112 of gestation, as well as on days 15 and 26 of lactation. The ultrasound scanner used in this study was equipped with an integrated manufacturer-provided calibration system. Prior to the start of the study and periodically during data collection, the device calibration was verified following the manufacturer’s instructions to ensure measurement accuracy and repeatability. Sow body weight was not recorded in this study; BFT and LMD were used as validated proxies of body condition and protein reserves.

#### 2.3.2. Sow Plasma Metabolites

To determine the presence of ketone bodies in blood, β-hydroxybutyrate (BHBA; mmol/L) was measured using the Freestyle Precision Beta-Ketone e system with B-ketone test strips (Abbott Laboratories, Chicago, IL, USA). This measurement was performed in both groups after 107 days of gestation and 26 days of lactation, with a value of 0 indicating a negative result and values greater than 0 indicating a positive result. The difference between BHBA after 26 days of lactation and BHBA after 107 days of gestation was calculated within individuals to assess BHBA evolution.

To determine muscle damage and sow catabolism and correlate these findings with BHBA levels, blood creatinine (CREA) concentrations were analyzed after 107 days of gestation. Blood samples were collected from sows and sent to a laboratory for biochemical analysis.

#### 2.3.3. Clinical Hypophagia

Clinical hypophagia (HP) was visually observed during the first week of lactation. Clinical hypophagia was assessed based on daily visual inspection of feed intake by trained farm staff. A sow was classified as hypophagia-positive when more than 50% of the offered ration remained not eaten in the trough, based on observations from two consecutive feedings. This criterion was applied as a practical and operational threshold under commercial farm conditions, where precise individual feed intake measurements were not feasible.

#### 2.3.4. Litter and Piglet Production Parameters

Farrowing was strictly monitored from start to finish to record the total number of piglets born (TB), including those alive born (AB) and stillborn (SB). Individual piglet weights were recorded at birth (before colostrum intake) and at 15 days of age. Litters presenting diarrhea during the first week of lactation were visually assessed and recorded. A litter was considered positive when clinical signs of diarrhea were observed in at least one piglet, whereas litters without any observable diarrheal symptoms were classified as negative.

### 2.4. Statistical Methods

Statistical analyses were performed using SPSS version 29 software (IBM, Chicago, IL, USA). For continuous variables with a single measurement per individual, a one-way analysis of variance (ANOVA) was used to compare means, with the group serving as the factor. This technique was also used to determine the relationship between a continuous and a categorical variable. The Shapiro–Wilk test and graphical methods (Q-Q plot) were used for checking residuals for normality. For quantitative variables with marked discontinuities, the Mann–Whitney U test (a non-parametric test) was applied. When a variable was measured repeatedly in the same individual at different times, a two-way mixed ANOVA was used, considering the group, time, and group × time interaction as factors. The relationships between two continuous variables were studied using Spearman’s correlation coefficient (rho). The strength of the association was small if 0.1 < |rho| < 0.3, medium/moderate when 0.3 < |rho| < 0.5, and large/strong when |rho| > 0.5. Linear regression was applied to understand the effect of BHBA on CREA after 107 days of gestation. For assessing the relationships between categorical variables, Pearson’s chi-squared test was used. In all cases, *p*-values < 0.050 were considered statistically significant. When a significant difference was found, effect size was estimated using Cohen’s d (quantitative variables) and ϕ or Cramer’s V (categorical variables). The effect was considered small for d < 0.500, medium for d = 0.500, and large for d > 0.500. Thresholds for ϕ were as follows: 0.100 (small), 0.300 (medium), and 0.500 (large). For Cramer’s V, thresholds were adjusted by degrees of freedom (df); for df = 2, the thresholds were 0.070 (small), 0.210 (medium), and 0.350 (large). When significant differences were detected in more than two variables, the Bonferroni correction for multiple comparisons was applied.

## 3. Results

### 3.1. Litter Characteristics

Table 2 shows the litter characteristics. Regarding the percentage of SB piglets in TB/litter, highly significant differences were observed between the groups, with a higher mean in group C (*p* = 0.001). The low stillbirth rates likely reflect the high health status of the herd, close farrowing supervision, and standardized management practices. These factors may have contributed to overall lower stillbirth percentages compared with industry averages.

For the percentage of piglets weighing less than 1 kg at birth in BA/litter, no significant differences were observed between the groups (*p* = 0.275). The mean piglet birth weight/litter significantly differed between the groups, with a higher mean in group T (*p* = 0.011). After 15 days of lactation, highly significant differences in mean piglet weight/litter were observed between the groups, with a higher mean in group T (*p* < 0.001). The number of piglets at 15 days after birth/litter showed significant differences between groups (*p* = 0.001); values were higher in group T. The effect size of treatment was small for the number of piglets at 15 days, medium for mean piglet birth weight/litter, and high for SB piglets in TB/litter and particularly for mean piglet weight/litter at 15 days of lactation.

In terms of neonatal diarrhea in piglets during the first week of lactation, highly significant differences were detected between the groups, with a lower frequency of diarrhea per litter observed in group T (*p* < 0.001). The effect of treatment can be considered large.

### 3.2. Sow Performance

The results for sow performance are shown in Table 3.

For BFT measurements at 80 and 112 days of gestation and at 15 days of lactation, no significant differences were detected between groups. However, at 26 days of lactation, highly significant differences were observed, with higher mean values in group T (*p* < 0.001); the effect of the treatment was large. For LMD, no significant differences were found between groups at any moment (*p* = 0.108). Regarding BFT measurements, both group C and group T showed highly significant differences across time points (*p* < 0.001). No significant differences in BFT were detected between 80 and 112 days of gestation, but from this point onwards, a significant decline in BFT values was observed at 15 and 26 days of lactation. Regarding the measurement of LMD, significant differences were detected between time points (*p* < 0.001); a significant decline in LMD values was observed in both groups from the first measurement to the last.

Regarding hypophagia in sows during the first week of lactation, significant differences were observed between the groups, with a lower frequency of hypophagia in group T (*p* = 0.014). The effect of the treatment can be considered small.

### 3.3. Sow Plasma Metabolites

Table 4 shows the plasma metabolite results for the studied sows. For CREA levels at 107 days of gestation, highly significant differences were also found between groups, with group C showing higher mean values (*p* < 0.001); the effect size of treatment was large.

**Figure 1:**
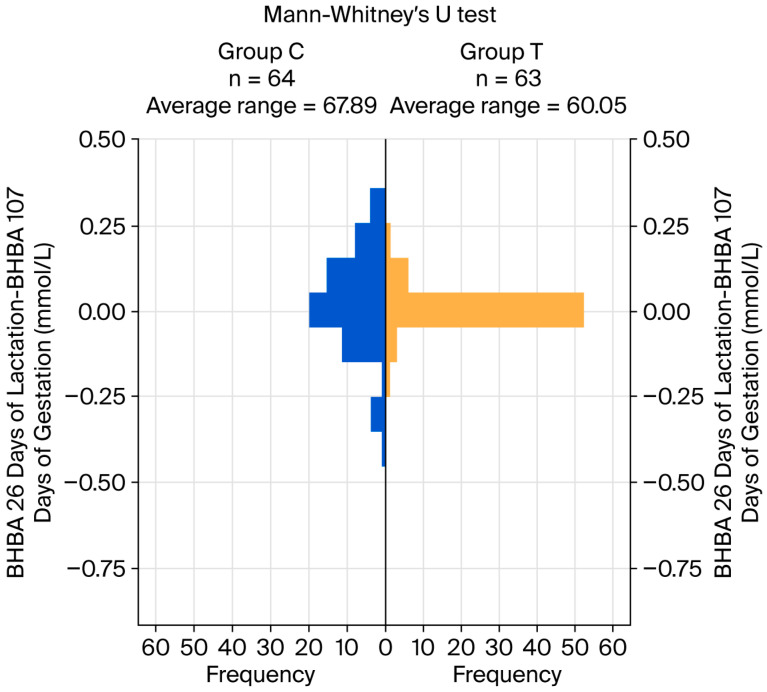
Pyramid chart for the differences in BHBA at 26 days of lactation and BHBA at 107 days of gestation (within individuals).

Regarding BHBA, highly significant differences were detected between groups at both 107 days of gestation and 26 days of lactation, with higher mean values observed in group C (*p* < 0.001). The effect size of the treatment was large at both moments. The within-individual difference between BHBA concentrations at both considered moments was used to assess the temporal evolution of this variable; no significant difference was detected between groups. Figure 1 shows a pyramid chart for the Mann–Whitney test, representing the distribution of these differences.

The distribution of the positive, negative, and 0 values for these within-individual differences showed a significant difference between groups. Group T showed a lower frequency of both positive and negative differences, with a higher frequency of differences equal to 0, with a large effect size of treatment (see Table 5; *p* < 0.001).

### 3.4. Relationships Between Sow Plasma Metabolites and Body Condition, and Litter and Piglet Characteristics and Hypophagia

The correlation coefficients for BHBA and CREA at 107 days of gestation, in relation to sow performance, are shown in Table 6.

A positive, significant, and medium correlation was found for BHBA and CREA at 107 days of gestation. A linear regression significantly related BHBA and CREA at 107 days of gestation (F (1,125) = 6.881, *p* = 0.010); the regression equation was CREA = 2.23 + 2.42 × BHBA. However, BHBA at 107 days of gestation only accounted for 5.2% variability in CREA at 107 days of gestation. At 107 days of gestation, both blood BHBA and CREA levels showed significant negative correlations with LMD at 112 days of gestation; the string of these relationships was small. The detected correlation between LMD at 112 days of gestation and CREA levels at 107 days of gestation may be driven by the interrelationship between CREA and BHBA values at 107 days of gestation.

The correlation coefficients for BHBA at 26 days of lactation, in relation to sow performance and litter characteristics, are shown in Table 7. Significant correlations were only found for BFT at 26 days of lactation, LMD at both 15 and 26 days of lactation, and mean piglet weight at 15 days of lactation/litter. In all cases, the correlations were negative and small.

Table 8 shows the relationships between metabolite concentrations and body conditions with the presence or absence of hypophagia and neonatal diarrhea.

The presence of hypophagia was significantly associated with higher concentrations of BHBA and CREA, with large effect sizes. Furthermore, low LMD values were significantly associated with hypophagia, although there were only medium effect sizes. An association was also identified between high BHBA and CREA levels and neonatal diarrhea; the effect size was large for both BHBA and CREA at 107 days of gestation and small for BHBA at 26 days of lactation.

Additionally, significant associations were detected between low values of LMD and the presence of hypophagia (large effect sizes). However, only low values of LMD at 112 days of lactation were significantly related to neonatal diarrhea with a small effect size. Also, significant relationships for neonatal diarrhea and low values of BFT at both 15 and 26 days of lactation were found; the size effects were small for BFT at 15 days of lactation and large for BFT at 26 days of lactation.

## 4. Discussion

The present study evaluated the effects of a targeted peripartum multi-nutrient supplementation strategy on metabolic adaptation, reproductive performance, and early litter growth in hyperprolific nulliparous sows under commercial conditions [40,41,42]. Overall, supplementation during late gestation and early lactation was associated with improved farrowing outcomes, a more favorable metabolic profile, better preservation of maternal body reserves, and enhanced early piglet performance [40,42,43,44].

Importantly, the effects observed in this study must be interpreted as responses to the complete nutritional supplement rather than to individual nutrients. The supplement provided additional energy, protein, amino acids, minerals, and metabolic cofactors during a physiologically critical period, and the experimental design does not allow the contribution of specific components to be isolated. Accordingly, the interpretation of the results focuses on the integrated nutritional effect of the supplement on maternal metabolism and reproductive performance, as previously emphasized for transition feeding strategies in modern sows [40,42,45,46].

A significant reduction in stillbirth rate was observed in supplemented gilts. This improvement likely reflects enhanced metabolic preparedness for parturition rather than the action of a single nutrient. Farrowing is an energetically demanding process requiring sustained uterine contractility and adequate ATP availability. Improving overall nutrient supply during late gestation may support more efficient uterine function, shorten farrowing duration, and reduce fetal hypoxia, thereby lowering stillbirth incidence, as reported in hyperprolific sow systems [41,47,48,49]. Although calcium availability and improved energy metabolism may plausibly contribute to these effects, such mechanisms should be regarded as complementary rather than independently causal.

Piglet birth weight and body weight at 15 days of lactation were significantly higher in the treatment group. These findings are consistent with improved maternal nutrient availability during late gestation and early lactation, which supports fetal growth and milk production [42,43,50,51]. An increased supply of energy and amino acids during late gestation has been shown to enhance placental efficiency and fetal nutrient transfer [50,52,53,54]. Rather than attributing these responses to specific amino acids or micronutrients, the present results suggest that supplementation corrected a marginal nutrient supply during a period of rapidly increasing fetal demands, as described for late-gestation nutritional strategies in gilts [40,42,44].

The lower incidence of neonatal diarrhea observed in the supplemented group further supports the relevance of maternal nutritional status during the peripartum period [42,55,56]. Heavier piglets at birth and during early lactation generally exhibit improved thermoregulation, greater colostrum intake, and enhanced immune competence, which collectively reduce susceptibility to enteric disorders [57,58,59]. However, the absence of direct measurements of colostrum yield, immunoglobulin concentration, or immune parameters represents a limitation of the present study. Consequently, the association between supplementation and reduced neonatal diarrhea should be interpreted as indirect and hypothesis-generating rather than causal, in line with previous observations on maternal nutrition and neonatal health [42,55,58].

A significantly lower prevalence of postpartum hypophagia was observed in the treatment group. Hypophagia during early lactation is closely linked to a negative energy balance and impaired metabolic adaptation [42,60,61]. In this study, control gilts exhibited higher concentrations of β-hydroxybutyrate (BHBA) at the end of gestation, indicating greater lipid mobilization prior to farrowing [60,62]. These results suggest that supplementation improved energy availability or utilization during late gestation, facilitating a smoother metabolic transition into lactation [40,42,43,62].

Finally, the generalizability of these findings should be interpreted with caution. This study was conducted on a single commercial farm under specific genetic, management, and environmental conditions. Nevertheless, the metabolic challenges addressed—large litter size, limited feed intake capacity, and peripartum metabolic stress—are common across modern hyperprolific sow systems [40,41,42,49,60]. This suggests that the observed benefits of targeted peripartum supplementation may be relevant to similar production contexts, although further studies under diverse conditions are warranted.

Overall, the present results demonstrate that improving overall nutrient supply during the peripartum period can positively influence metabolic adaptation, reproductive efficiency, and early litter performance in hyperprolific nulliparous sows [40,41,42,43,44]. While the contribution of individual nutrients cannot be isolated, the findings provide practical evidence that multi-nutrient supplementation during late gestation and early lactation represents an effective strategy to support metabolic resilience under commercial production conditions [40,42,45,46,60].

## 5. Conclusions

The present study demonstrates that targeted multi-nutrient supplementation during the peripartum period can positively influence metabolic adaptation, reproductive performance, and early litter outcomes in hyperprolific nulliparous sows under commercial conditions. Supplementation during late gestation and early lactation was associated with reduced stillbirth rates, improved metabolic stability, greater preservation of maternal body reserves, and enhanced early piglet growth.

The observed benefits are best interpreted as the result of improved overall nutrient availability during a critical physiological window, rather than the action of individual nutrients. Associations between metabolic indicators, including β-hydroxybutyrate, creatinine, and longissimus muscle depth, and productive outcomes highlight the close coupling between energy balance, protein mobilization, and lactational performance in hyperprolific gilts.

From a practical perspective, targeted peripartum supplementation represents a feasible nutritional strategy for commercial herds characterized by large litter sizes and a limited voluntary feed intake capacity. Although a formal economic evaluation was beyond the scope of this study, the improvements observed in reproductive outcomes and early piglet performance suggest potential cost–benefit advantages that merit further investigation.

Overall, these findings provide applied evidence that supporting metabolic resilience through multi-nutrient supplementation during the peripartum period may contribute to improved productivity and animal robustness in modern hyperprolific sow systems.

## Figures and Tables

**Table 1:** Composition of the diets, gestation standard, and lactation standard of both groups C and T.

Item (Unit)		Gestation Standard	Lactation Standard
Ingredients	Barley (%)	48.410	22.030
	Maize (%)	0.000	19.440
	Wheat (%)	0.000	0.000
	Soybean meal (47% crude protein)	0.000	18.900
	Animal blended fat (%)	0.000	0.000
	Beet pulp (%)	0.000	0.000
	Full-fat soya (%)	0.000	0.000
	Rice cylinder (%)	10.000	5.000
	By-product biscuit (%)	0.000	7.000
	Zootechnical meal (%)	7.430	10.000
	Sodium bicarbonate (%)	0.100	0.310
	Monocalcium phosphate (%)	0.090	0.580
	Dicalcium phosphate (%)	0.000	0.000
	Salt (%)	0.400	0.150
	L-Lisine 50 (%)	0.330	0.270
	L-Threonine (%)	0.040	0.050
	L-Methionine (%)	0.000	0.000
	Choline chloride (%)	0.050	0.040
	Soybean oil (%)	0.500	1.480
	Calcium carbonate (%)	1.620	1.720
	Sunflower meal (36% crude protein)	10.000	2.700
	Wheat bran (%)	18.980	10.000
	Alfalfa (%)	0.480	0.000
	Sepiolite (%)	1.320	0.000
	Glucogenic precursor (%)	0.000	0.000
	Others (%, additives) ^1^	0.240	0.300
Nutrients ^2^	Metabolizable energy (kcal/kg)	2844.879	3174.143
	Fat matter (%)	4.106	5.008
	Crude protein (%)	12.500	17.000
	Crude fiber (%)	8.000	5.000
	Neutral detergent fiber (%)	23.460	15.953
	Arginine (%)	0.812	1.108
	Digestible arginine (%)	0.672	0.978
	Lysine (%)	0.650	0.973
	Digestible lysine (%)	0.508	0.811
	Methionine (%)	0.215	0.284
	Digestive methionine (%)	0.176	0.243
	Methionine + cysteine (%)	0.479	0.587
	Methionine + cysteine digestive (%)	0.358	0.463
	Calcium (%)	0.900	1.000
	Phosphorus, Total (%)	0.616	0.613
	Phosphorus, Digestive (%)	0.230	0.340

^1^ Choline, phytases, antioxidants, mycotoxin sequestrant. ^2^ Calculated composition according to FEDNA [38].

**Table 2:** Results for litter characteristics in both groups. Data are count/n (percentage) or mean ± SD (standard deviation). Effect size was estimated using Cohen’s d (quantitative variables) or ϕ (categorical variables).

Variable	Group C		Group T		*p*-Value	Effect Size
	n	Mean ± SD; Count/n (%)	n	Mean ± SD; Count/n (%)		
Total born piglets/L	64	16.92 ± 3.739	63	16.03 ± 3.422	0.147	
Alive born piglets/L	64	16.11 ± 3.591	63	15.75 ± 3.336	0.571	
Stillborn piglets/L (%)	64	4.63 ± 5.512	63	1.80 ± 4.013	0.001	0.587
Piglets weighing less than 1 kg at birth/L	64	13.02 ± 10.861	63	11.43 ± 11.739	0.275	
Mean piglet birth weight/L (kg)	60	1.19 ± 0.151	54	1.27 ± 0.174	0.011	0.491
Mean piglet weight at 15 days of lactation/L (kg)	60	3.25 ± 0.486	54	3.62 ± 0.542	<0.001	0.719
Piglets at 15 days after birth/L	60	11.90 ± 2.129	54	12.59 ± 1.108	0.001	0.406
Neonatal diarrhea in piglets during the first week of lactation/L		39/64 (60.9%)		8/63 (12.7%)	<0.001	0.500

**Table 3:** Results for sow performance in both groups. Data are count/n (percentage) or mean ± SD (standard deviation). ^a,b,c,d^: Different letters indicate significant differences between moments within group (column). Effect size was estimated using Cohen’s d (quantitative variables) or ϕ (categorical variables).

Variable		Group C (n = 64)	Group T (n = 63)	Effect (*p*-Value)		Effect Size (Group)
		Mean ± SD (mm); Count/n (%)	Mean ± SD (mm); Count/n (%)	Group x Moment	Group	
BFT	80 days of gestation	14.06 ± 3.576 ^a^	13.87 ± 3.177 ^a^	<0.001	0.744	
	112 days of gestation	13.60 ± 3.265 ^a^	13.50 ± 2.673 ^a^		0.848	
	15 days of lactation	11.92 ± 3.078 ^b^	12.40 ± 2.227 ^b^		0.315	
	26 days of lactation	10.02 ± 2.322 ^c^	11.46 ± 2.069 ^c^		<0.001	0.655
	Moment Effect (*p*-value)	<0.001	<0.001			
LMD	80 days of gestation	49.21 ± 5.668 ^a^	49.15 ± 4.382 ^a^	0.145	0.108	
	112 days of gestation	47.35 ± 5.647 ^b^	48.53 ± 5.368 ^b^			
	15 days of lactation	45.41 ± 6.173 ^c^	47.03 ± 5.017 ^c^			
	26 days of lactation	44.09 ± 6.381 ^d^	46.28 ± 4.492 ^d^			
	Moment Effect (*p*-value)	<0.001				
Hypophagia in sows during the first week of lactation		15/64 (23.4%)	4/63 (6.3%)		0.014	0.240

**Table 4:** Results for sow plasma metabolites in both groups. Data are mean ± SD (standard deviation). Effect size was estimated using Cohen’s d.

Variable	Group C (n = 64)	Group T (n = 63)	*p*-Value	Effect Size
	Mean ± SD	Mean ± SD		
CREA after 107 days of gestation (mmol/L)	2.794 ± 1.4283	2.071 ± 0.6917	<0.001	0.644
BHBA after 107 days of gestation (mmol/L)	0.131 ± 0.1283	0.040 ± 0.0636	<0.001	0.899
BHBA after 26 days of lactation (mmol/L)	0.153 ± 0.1391	0.044 ± 0.0690	<0.001	0.993
BHBA after 26 days of lactation–BHBA after 107 days of gestation (mmol/L)	0.022 ± 0.1527	0.005 ± 0.0521	0.183	

**Table 5:** Distribution of positive, negative, and 0 values for the within-individual differences between BHBA after 26 days of lactation and BHBA after 107 days of gestation (mmol/L). Data are count/n (%). ^a,b^: Different letters indicate significant differences between groups (column). Effect size was estimated using Cramer’s V.

BHBA After 26 Days of Lactation–BHBA After 107 Days of Gestation (mmol/L)	Group C; Count/n (%)	Group T; Count/n (%)	*p*-Value	Effect Size
Increases (difference > 0)	27/64 (42.2%) ^a^	7/63 (11.1%) ^b^	<0.001	0.518
Decreases (difference < 0)	17/64 (26.6%) ^a^	4/63 (6.3%) ^b^		
No changes (difference = 0)	20/64 (31.3%) ^a^	52/63 (82.5%) ^b^		

**Table 6:** Correlation coefficients for the concentration of BHBA and CREA at 107 days of gestation with sow performance.

Variable 1 (n = 127)	Variable 2 (n = 127)					
	CREA at 107 Days of Gestation (mmol/L)		BFT at 112 Days of Gestation (mm)		LMD at 112 Days of Gestation (mm)	
	Rho	*p*-Value	Rho	*p*-Value	Rho	*p*-Value
BHBA at 107 days of gestation (mmol/L)	0.456	<0.001	−0.167	0.061	−0.217	0.014
CREA at 107 days of gestation (mmol/L)			−0.131	0.142	−0.285	0.001

**Table 7:** Relationships between the concentration of BHBA after 26 days of lactation with sow performance and litter.

Variable	BHBA After 26 Days of Lactation (mmol/L)	
	Rho	*p*-Value
BFT at 15 days of lactation (n = 127)	−0.147	0.099
BFT at 26 days of lactation (n = 127)	−0.212	0.017
LMD at 15 days of lactation (n = 127)	−0.357	<0.001
LMD at 26 days of lactation (n = 127)	−0.268	0.002
Piglets weighing less than 1 kg at birth/L (n = 127)	0.012	0.896
Stillborn piglets/L (%; n = 127)	0.115	0.196
Mean piglet birth weight/L (kg; n = 127)	−0.125	0.162
Piglets at 15 days after birth (n = 114)	−0.178	0.059
Mean piglet weight at 15 days of lactation/litter (kg; n = 114)	−0.208	0.026

**Table 8:** Relationships between metabolite concentrations and body conditions with the presence or absence of hypophagia and neonatal diarrhea.

Variable	Hypophagia				Neonatal Diarrhea			
	Absence (n = 108)	Presence (n = 19)	*p*-Value	Effect Size	Absence (n = 80)	Presence (n = 47)	*p*-Value	Effect Size
	Mean ± SD	Mean ± SD			Mean ± SD	Mean ± SD		
BHBA at 107 days of gestation (mmol/L)	0.071 ± 0.0967	0.168 ± 0.1493	<0.001	0.771	0.061 ± 0.0879	0.128 ± 0.1330	0.001	0.594
BHBA at 26 days of lactation (mmol/L)	0.085 ± 0.1118	0.179 ± 0.1512	0.004	0.693	0.081 ± 0.1092	0.130 ± 0.1382	0.036	0.393
CREA at 107 days of gestation (mmol/L)	2.405 ± 1.235	2.609 ± 0.774	0.488		2.212 ± 0.7690	2.815 ± 1.5967	0.005	0.481
BFT 112 days of gestation (mm)	13.46 ± 2.868	14.05 ± 3.568	0.436		13.76 ± 2.968	13.18 ± 2.983	0.291	
BFT 15 days of lactation (mm)	12.31 ± 2.538	11.27 ± 3.375	0.121		12.62 ± 2.495	11.36 ± 2.846	0.010	0.471
BFT 26 days of lactation (mm)	10.87 ± 2.186	9.95 ± 2.846	0.108		11.20 ± 2.183	9.93 ± 2.316	0.003	0.564
LMD 112 days of gestation (mm)	48.41 ± 5.518	45.628 ± 4.851	0.022	0.533	48.74 ± 5.349	46.57 ± 5.593	0.031	0.396
LMD 15 days of lactation (mm)	46.63 ± 5.595	43.83 ± 5.614	0.046	0.500	46.53 ± 5.338	45.67 ± 6.204	0.411	
LMD 26 days of lactation (mm)	45.61 ± 5.592	42.69 ± 5.190	0.036	0.541	45.67 ± 4.960	44.33 ± 6.547	0.194	

## Data Availability

The original contributions presented in this study are included in the article. Further inquiries can be directed to the corresponding author.

## Abbreviations

The following abbreviations are used in this manuscript:

ANOVA	analysis of variance
BA	born alive
BFT	backfat thickness
BHBA	b-hydroxybutyrate
BHT	Butylated hydroxytoluene
C	Control
CREA	creatinine
FDNA	Development of Animal Nutrition
HP	hypophagia
LMD	longissimus muscle depth
mEq	milliequivalents
SB	stillborn
SD	standard deviation
T	Treatment
TB	total born
